# Whole-Genome Resequencing of *Cucurbita maxima* and *Cucurbita moschata* Provides Insights into Genomic Variants Associated with Morphology and Quality Traits

**DOI:** 10.3390/ijms27114903

**Published:** 2026-05-28

**Authors:** Jian Wang, Jing Liu, Xiaohua Wu, Yu Huang, Ying Wang, Xuanhe Guo, Baogen Wang, Xiang Niu, Xinyi Wu, Lan Ding, Weizhong Huang, Guojing Li, Xiaojiang Xu

**Affiliations:** 1 A State Key Laboratory for Quality and Safety of Agro-Products, Key Laboratory of Vegetable Germplasm Innovation and Quality Breeding in the Province, Institute of Vegetables, Zhejiang Academy of Agricultural Sciences, Hangzhou 310021, China; wangjian@zaas.ac.cn (J.W.); ligj@zaas.ac.cn (G.L.); 2 Institute of Vegetables, Shaoxing Academy of Agricultural Sciences, Shaoxing 312003, China

**Keywords:** *Cucurbita*, genomic variation, fruit quality, leaf trait

## Abstract

The genus *Cucurbita* (pumpkin), encompassing *C. maxima* and *C. moschata*, is agriculturally and nutritionally significant globally. Herein, we re-sequenced 146 germplasm accessions (51 *C. moschata* and 95 *C. maxima*) to characterize genomic variations and identify loci associated with critical traits. Population structure analysis revealed four distinct subgroups: G1 (*C. moschata*), and G2–G4 (*C. maxima*), the latter classified by rind color (green, gray, and red, respectively). A genome-wide association study (GWAS) analysis identified 26 loci associated with eight fruit quality traits (e.g., rind color, pulp thickness, starch content) and leaf traits. Selective sweep analyses revealed 18 overlapping signals between the GWAS and selective regions, highlighting convergent evolution in starch content, pulp thickness, and water content driven by artificial and natural selection. We further validated these key fruit quality candidate genes, confirming that starch, cellulose, and pulp trait-related genes exhibited genotype-specific expression consistent with the quality divergence between CMO-X and CMO-E. Notably, *C. moschata* exhibited higher β-carotene and water content, while *C. maxima* showed higher starch content, reflecting divergent selection pressures. For leaf traits, 13 loci associated with leaf length were found, including LL9.2/LW9.1 with *Cmax09G001045,* which regulates leaf size. A novel haplotype in *Cmax09G001045* explained the small-leaf phenotype of ‘Cuili 5’. This study provides a comprehensive genomic variation map of *C. maxima* and *C. moschata*, clarifies the genetic bases of key agronomic traits, and sheds light on their domestication and selection history, offering valuable resources for molecular breeding and crop improvement.

## 1. Introduction

The genus *Cucurbita* (2n = 40), commonly known as pumpkin, holds multifaceted significance in global agriculture, nutrition, and industrial applications, positioning it as a critical yet underutilized crop with substantial potential for addressing contemporary challenges in food security and sustainable development. It originated in North and South America and spread to Europe, Asia and other continents following Columbus’ discovery of the New World [1]. China ranks first in global pumpkin production and consumption, with 7.42 million tons of production and over 0.4 million hectares of cultivation area in 2023, respectively (FAO, http://www.fao.org/faostat/en/#data, accessed on 16 May 2026). In this genus, the most widely cultivated and economically important species include *C. maxima*, *C. moschata* and *C. pepo*, of which the flowers, fruits, leaves and seeds are edible and used for oil extraction and medicinal purposes [2,3,4].

The two species, *C. maxima* (commonly known as winter squash) and *C. moschata* (often referred to as pumpkin) are predominantly cultivated in China and exhibit distinct morphological, ecological, and agricultural characteristics [5]. According to archeological data, *C. maxima* has its origin in Mesoamerica, while *C. moschata* hails from northern South America [1]. Subsequently, *C. maxima* experienced a significant process of diversification within its secondary domestication center spanning China and Japan, whereas *C. moschata* underwent the same in its India–Myanmar secondary domestication center [1,6]. They are rich in bioactive compounds, including antioxidants, vitamins (e.g., β-carotene), and minerals, which contribute to their role in combating malnutrition and chronic diseases [7]. They serve as a fundamental staple food in numerous developing nations and have a global consumption footprint. Beyond their applications in the culinary realm, the fruits of these crops are also employed as decorative items and carved into elaborate lanterns [8]. In addition, they are utilized as rootstocks for other cucurbit crops such as cucumber, melon and watermelon to enhance resilience against soil-borne pathogens and abiotic environmental stresses [9].

Although *C. maxima* and *C. moschata* have many similarities, they exhibit marked differences in fruit quality and sensory attributes, driven by their distinct biochemical compositions and morphological traits. *C. moschata* fruits are renowned for their dense, firm flesh with a fine, smooth texture, characterized by high dry matter content (15–25%) and elevated β-carotene levels, which impart a rich, sweet flavor and a vibrant orange hue. In contrast, *C. maxima* cultivars, such as the widely cultivated ‘Kabocha’ or ‘Red Kuri,’ display a drier, starchier flesh with a slightly granular mouthfeel, attributed to higher starch-to-sugar ratios and lower water activity [10,11]. Notably, *C. moschata* fruits exhibit superior storage durability due to thicker pericarp layers and waxy cuticles, maintaining flavor integrity for months post-harvest, whereas *C. maxima* varieties, particularly giant ornamental types, tend to degrade more rapidly in texture and sweetness. These qualitative disparities reflect divergent selection pressures: *C. moschata* was historically optimized for nutrient-dense, shelf-stable food sources in tropical agroecosystems, while *C. maxima* evolved under cooler highland conditions favoring carbohydrate-rich, structurally robust fruits [12]. Modern sensory analyses further confirm that *C. moschata* ranks higher in consumer preference for sweetness and smoothness, whereas *C. maxima* is preferred in contexts requiring textural stability during thermal processing.

Herein, we re-sequenced 146 pumpkin germplasms of *C. maxima* and *C. moschata* to characterize the genetic variations in pumpkins. In addition, GWAS and selective sweeps were carried out with the aim of pinpointing the genes responsible for vital agronomic traits. In our study, we uncovered a comprehensive view of the genome sequence variations existing between the two subspecies. This not only offers valuable insights into the domestication process of pumpkin, but also sheds light on how it has been improved under selection pressure.

## 2. Results

### 2.1. Population Structure and Genomic Variation in Pumpkin

To decipher the genetic variation in pumpkin, we re-sequenced 146 *C. maxima* and *C. moschata* accessions exhibiting various traits, achieving an average sequencing depth of 19.74× and generating 1.12 Tb raw data (Supplementary Table S1). Approximately 98.17% of the clean reads were mapped to the reference genome HZAU_1.0 [13]. Interestingly, although the reference genome was derived from a highly salt-tolerant inbred line of *C. maxima*, no differences in the mapping percentage were detected between *C. maxima* and *C. moschata*. Finally, we retrieved 10,489,610 high-quality SNPs and 1,982,096 InDels. A total of 1,278,300 SNPs and 27,561 InDels were identified in the coding regions. Based on their functions, 633,898 SNPs (49.6%) led to non-synonymous mutations, continued or early terminated transcripts while 9,197 InDels (33.4%) caused frameshift changes (Table 1 and Table 2). The number of SNP variations in each accession varied from 414,798 (R-14) to 4,149,104 (Z-18) while the number of InDels varied from 88,695 (R-14) to 828,474 (Z-15) (Supplementary Table S2).

### 2.2. A Circos Diagram Illustrating the Genome-Wide Variations Among 146 Accessions

**Figure 1:**
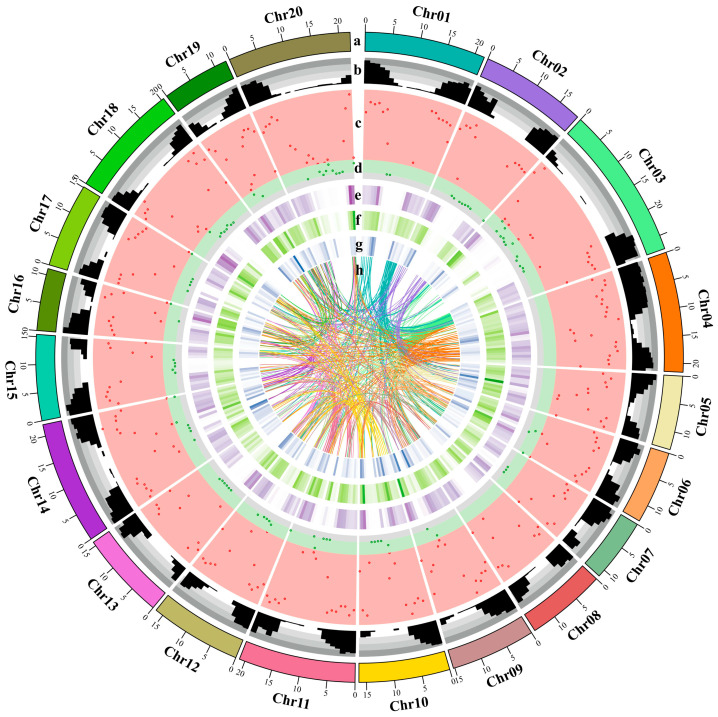
Genomic landscape of pumpkin. (**a**) Chromosome lengths (unit: Mb); (**b**) frequency histogram depicting gene density across chromosomal regions; (**c**) scatter plot illustrating SNP density > 0.0015 highlighted in red across chromosomal regions; (**d**) scatter plot displaying SNP density ≤ 0.0015 denoted by green points across chromosomal regions; (**e**) large deletions (DEL) mapped to chromosomal positions and visualized in purple; (**f**) insertion-type structural variations (INS) localized to chromosomal coordinates and displayed in green; (**g**) inversion-type structural variations (INV) annotated at chromosomal loci and represented in blue; (**h**) interchromosomal translocations (BND/CTX) identified between pairwise chromosomes. In all cases, quantitative tracks are aggregated in 1 Mb windows.

The distribution of SNPs along the chromosomes was not quite homogeneous (Figure 1). Each chromosome had a gap zone with very low gene density which was defined as the centromere (Figure 1b and Supplementary Figure S1a,b). A positive correlation was observed between gene density and SNP density, with a Pearson correlation coefficient of 0.89, indicating that gene-rich regions are associated with higher recombination rates. The average number of SNPs per Kb and genes per Mb were 31 and 84, respectively (Figure 1c,d and Supplementary Table S3). Large deletions, structural variations and interchromosomal translocations were located on chr01, chr05, chr09 (Figure 1e–g).

### 2.3. Genetic Diversity and Structure in the Whole Population

**Figure 2:**
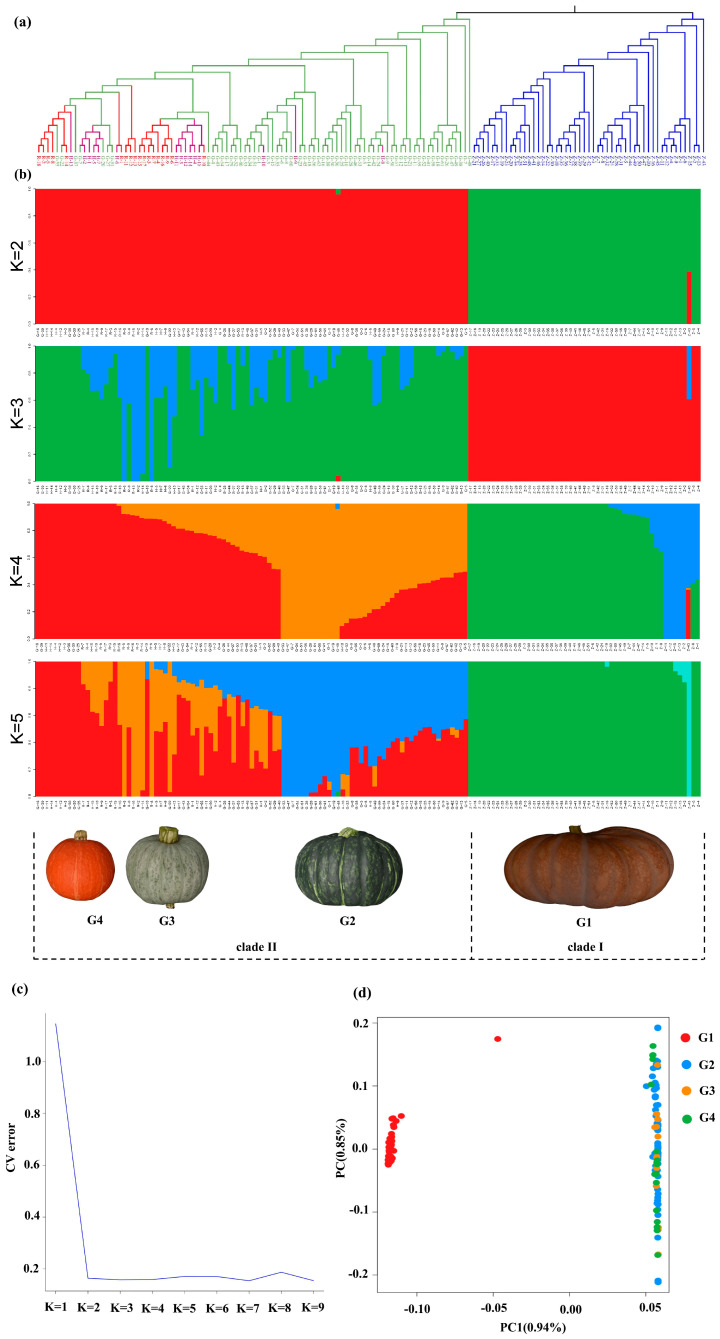
The population structure and genomic diversity of pumpkin accessions. (**a**) Neighbor-joining tree of 146 pumpkin accessions with different groups represented by different colors; (**b**) the structure model-based clustering with K from 2 to 5; (**c**) the line chart of cross-validation error rate; (**d**) PCA plots showing two divergent clades of 146 pumpkin accessions.

Subsequently, we analyzed the phylogenetic relationships of the 146 accessions. Genetic structure analysis for clusters (K) ranging from 2 to 5, combined with the coefficient of variation (CV) error plot, revealed that K = 4 was the optimal number of clusters, exhibiting the smallest CV error (Figure 2a,b). The PCA and phylogenetic graph validated four distinct subgroups as well (Figure 2c,d). Generally, the population of 146 accessions could be empirically divided into two ecotypes, depended on their differentiation of subspecies. The first clade (G1, n = 51) was composed entirely of *C. moschata*. The remaining 95 accessions of *C. maxima* were divided into three subpopulations: G2 (n = 62), G3 (n = 15) and G4 (n = 18), which could be roughly distinguished based on their rind color. The G2 subclade displayed a predominant green-skinned phenotype, whereas G3 and G4 consisted of gray-skinned and red-skinned accessions, respectively (Figure 2b).

**Figure 3:**
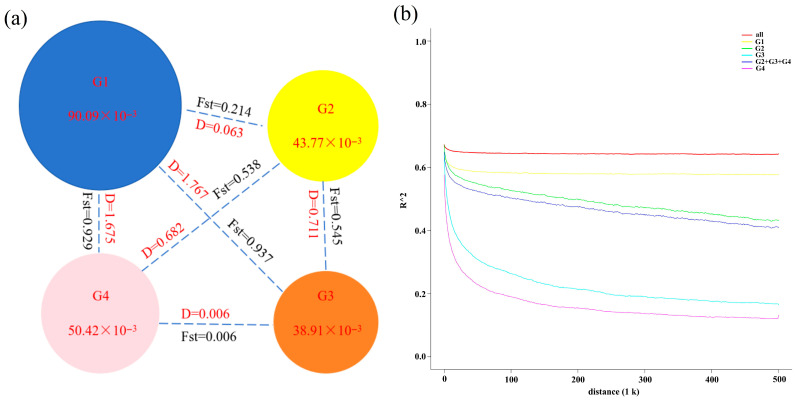
Genetic diversity and divergence of four pumpkin populations. (**a**) Nucleotide diversity (π), population differentiation fixation index (FST) and genetic distance (D) across the four groups. The value in each circle indicates π for each group. (**b**) LD decay plots of four group-specific populations.

The genetic diversity and evolutionary dynamics of the *Cucurbita* accessions—comprising the total population (all), *C. moschata* (Chinese pumpkin, G1), and the three *C. maxima* subgroups (G2, G3, and G4)—were evaluated using multiple genetic indices (Table 3). The total population of all accessions exhibited the highest genetic diversity, with the highest PIC (0.313). The expected heterozygosity (He = 0.396) and Shannon’s Information Index (I = 0.622) further confirmed its rich allelic variations. However, the low observed heterozygosity (Ho = 0.023) and extreme inbreeding coefficient (Fis = 0.91) suggested severe inbreeding, likely due to population bottlenecks and limited gene flow between differentiated subgroups. The positive Tajima’s D (0.235) implied balancing selection or population structure maintaining intermediate-frequency alleles. A moderate inbreeding coefficient (Fis = 0.583) and a negative Tajima’s D (−0.108) in *C. moschata* indicated restricted mating and potential recent population expansion or purifying selection, possibly linked to domestication. The *C. maxima* subgroups (G1, G2, G3) displayed critically low diversity and high inbreeding (Fis = 0.74–0.77). The strongly negative Tajima’s D suggested directional selection (e.g., trait-specific breeding) or demographic contraction. The *C. moschata* group showed reduced diversity, influenced by inbreeding and domestication-related selection, while *C. maxima* faced severe genetic erosion, driven by intense inbreeding and potential bottlenecks. The genetic differentiation coefficients of the four populations also demonstrated this; the pairwise population differentiation fixation index (FST) varied from 0.006 to 0.937 (Figure 3a). Furthermore, the values of FST and genetic distance (D) showed a positive correlation. G1 had the highest nucleotide diversity (π) while G3 exhibited the lowest π indicating that G3 had a relatively narrow genetic background. The order of the decay rates of LD was G4 > G3 > G2 + G3 + G4 > G2 > G1 > all populations (Figure 3b). It also verified a stronger selective pressure and a higher degree of domestication in *C. maxima* than in *C. moschata* from another perspective.

### 2.4. GWAS in Fruti Quality Traits

**Figure 4:**
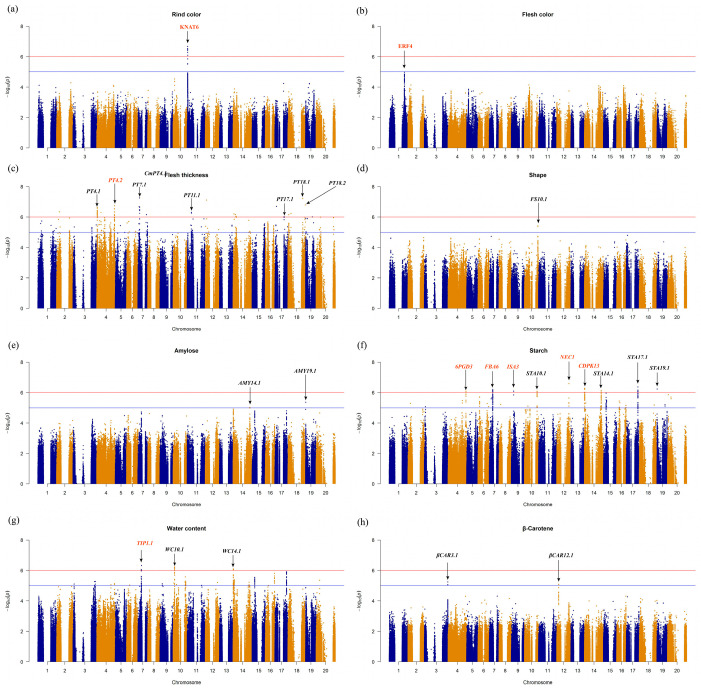
GWAS of pumpkin fruit quality traits. (**a**–**h**), Manhattan plots of GWAS of rind color (**a**), pulp color (**b**), pulp thickness (**c**), fruit shape (**d**), amylose (**e**), starch (**f**), water content (**g**) and β-carotene using the pumpkin population. Significant GWAS signals are indicated by vertical black arrows. Known QTL regions or candidate genes are marked in red. Blue and red horizontal solid lines indicate the Bonferroni-corrected significance thresholds of GWAS (α = 0.05 and α = 0.01, respectively). KNAT6, knotted-1-like 6; ERF4, ethylene-responsive transcription factor 4; 6PGD3, 6-phosphogluconate dehydrogenase, decarboxylating 3; FBA6, fructose-bisphosphate aldolase 6; ISA3, isoamylase 3; NEC1, nectary tissue expressed gene; CDPK13, calcium-dependent protein kinase 13; TIP1.1, tonoplast intrinsic protein 1-1.

We used 4.21 million high-quality SNPs in the GWAS analysis in order to identify remarkable SNPs associated with fruit quality traits. Four different statistical programs were performed and compared in GLM, GLM with Q-matrix (GLMQ), MLM with K-matrix (MLMk) and MLM with both K and Q matrices (MLMkq) models. The MLMk model provided the best fit to the data, identifying 26 loci significantly associated with eight quality traits including the appearance quality (rind color, pulp color, pulp thickness and fruit shape) and the inner quality (content of amylose, β-carotene, water and starch) (Figure 4). Loci were considered significant at a genome-wide threshold of *p* < 1 × 10^−5^, with Bonferroni correction applied. Within the previously reported quantitative trait loci (QTL) *CmaMg*, a peak demonstrating a strong association with rind color was identified on chromosome 11, as well [14]. This QTL harbors the homeobox protein knotted-1-like 6 gene *CmaKNAT6* (*Cmax11G000090*; Figure 4a). Another genomic region exhibiting a robust association with pulp color was detected on chromosome 1 (Figure 4b). This region contained ethylene-responsive transcription factor 4 (ERF4) gene *Cmax01G001188*, which has been found to synergistically reduce the expression of target genes involved in anthocyanin biosynthesis [15]. Seven signals significantly associated with pulp thickness were identified on chromosomes 4, 7, 11, 17 and 18 (Figure 4c), with one QTL *PT4.2* overlapping with the pulp color QTL *fft4.1* detected from recombinant inbred lines (RILs) of *C. maxima* [16]. A total of 9 signals associated with starch content were identified. Among these, five prominent signals were located in close proximity to the *6PGD3* (*Cmax04G002570*), *FBA6* (*Cmax07G000787*), *ISA3* (*Cmax09G000487*), *NEC1* (*Cmax12G000875*), and *CDPK13* (*Cmax14G000051*) genes respectively, which have been previously established to regulate important precursors for starch synthesis [17,18], structural modification and metabolic process of starch granules [19,20], transportation of sugars between cells [21], the metabolism of starch [22,23] (Figure 4f). Three peaks exhibiting a high degree of association with water content were detected on chromosomes 7, 10, and 14 (Figure 4g). Candidate genes in these peaks included tonoplast intrinsic protein *TIP1.1* (*Cmax07G000804*), which encodes an aquaporin that functions as a water channel [24]. In total, 1, 2 and 2 signals were found to be associated with fruit shape, amylose content and β-carotene content, respectively (Figure 4d,e,h). No obvious candidate genes were found in the QTL of fruit shape, amylose and β-carotene. In summary, eight putative genes related to fruit quality traits were predicted through GWAS analysis (Supplementary Table S4).

### 2.5. Evolution and Domestication of Fruit Quality Traits

**Figure 5:**
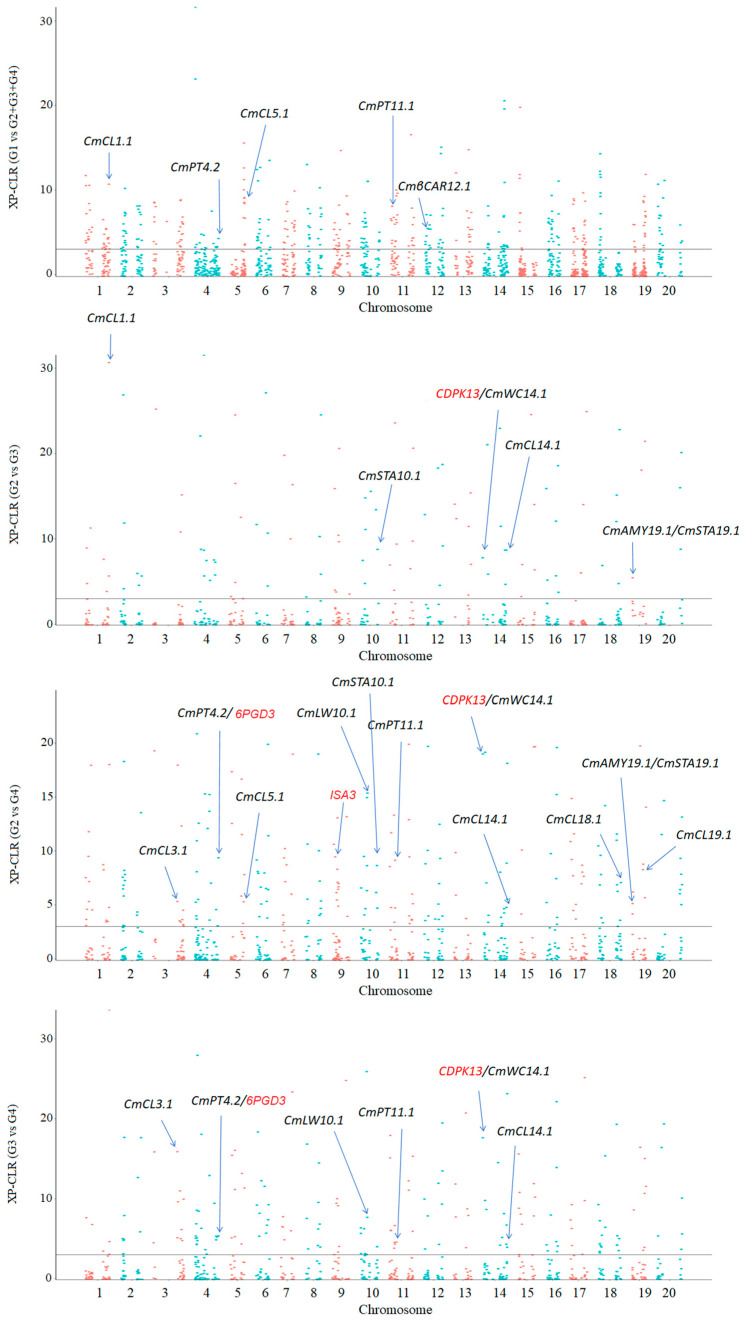
Genome-wide distribution of selective sweeps through four different comparisons. QTL regions are shown in black text. Candidate genes in the selective regions are indicated in red text. CL: cellulose; PT: pulp thickness; βCAR: β-carotene content; STA: starch content; WC: water content; AMY: amylose; LW: leaf width; 6PGD3: 6-phosphogluconate dehydrogenase, decarboxylating 3; ISA3: isoamylase 3; CDPK13: calcium-dependent protein kinase 13.

To delineate how human and natural selection have shaped the different fruit quality types in pumpkin, we further searched for signatures of selection in the pumpkin genome by comparing the selective sweeps among the four subgroups. Through XP-CLR analyses, we identified 394, 109, 225 and 186 potential selective loci in the G1/(G2 + G3 + G4), G2/G3, G2/G4, and G3/G4 comparisons, respectively (Supplementary Table S5). A total of 18 signals related to cellulose content (CL), pulp thickness (PT), β-carotene content (βCAR), starch content (STA), water content (WC), amylose content (AMY) and leaf width (LW) were jointly identified through selective sweep detection and GWAS analyses (Figure 4 and Figure 5, Supplementary Table S6). Among these comparisons, G2/G4 had the greatest number of significant signals (14 loci) while the other three comparisons had similar numbers of identified signals, indicating that the fruit quality traits between G2 and G4 might have undergo more stringent artificial selection (Figure 5). Intriguingly, there were three pairs of overlapping selective sweeps identified in the same regions. The putative gene *CDPK13* (co-localized with *CmWC14.1*), and *CmAMY19.1* (co-localized with *CmSTA19.1*) were detected twice in both G2/G3 and G2/G4. The putative gene *CmPT4.2*, which overlapped with the *6PGD3* gene, was simultaneously identified in both G2/G4 and G3/G4 comparisons. As expected, *CmAMY19.1* linked to amylose was closely associated with *CmSTA19.1* linked to starch (Figure 5). The remaining overlapping selective sweeps *CDPK13*/*CmWC14.1* and *CmPT4.2/6PGD3* implied that starch content, pulp thickness, and water content in fruits underwent convergent evolution driven by artificial and natural selection in some aspects.

**Figure 6:**
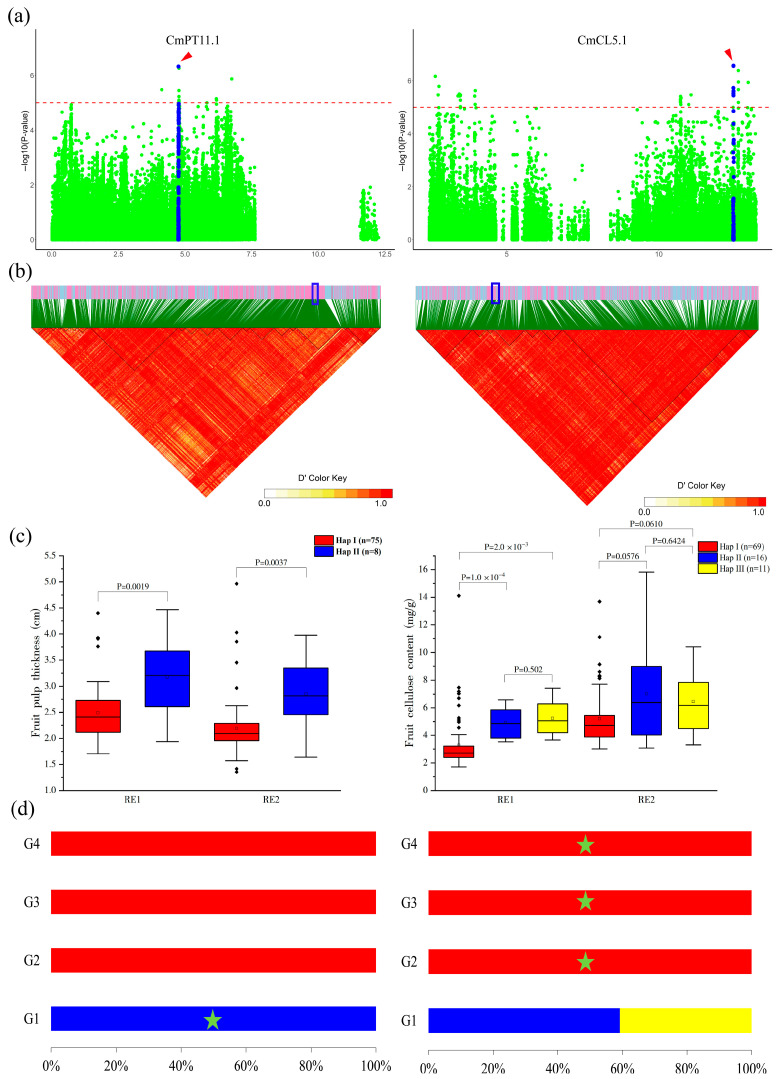
The repeatedly identified signals of selective sweeps and GWAS. (**a**) Local Manhattan plot for CmPT11.1 and CmCL5.1; (**b**) local LD block analysis for CmPT11.1 and CmCL5.1; (**c**) haplotype analysis for CmPT11.1 and CmCL5.1; (**d**) the distribution in different pumpkin subpopulations for CmPT11.1 and CmCL5.1. Putative genes associated with each signal are highlighted in blue boxes within the LD heatmaps. For haplotype analysis, the “n” values in histograms and boxplots represent the number of accessions carrying the corresponding haplotypes. In boxplots, the lower and upper edges of boxes indicate the 25% and 75% quartiles, respectively; central lines denote medians, and small hollow squares mark means. Whiskers extend to 1.5× the interquartile range, with small solid diamonds indicating outliers. *p*-values from two-sided Student’s *t*-tests are displayed above the boxplots. The olive-green star indicates the haplotypes related to a higher fruit pulp thickness and cellulose content, respectively (from (**left**) to (**right**)).

**Figure 7:**
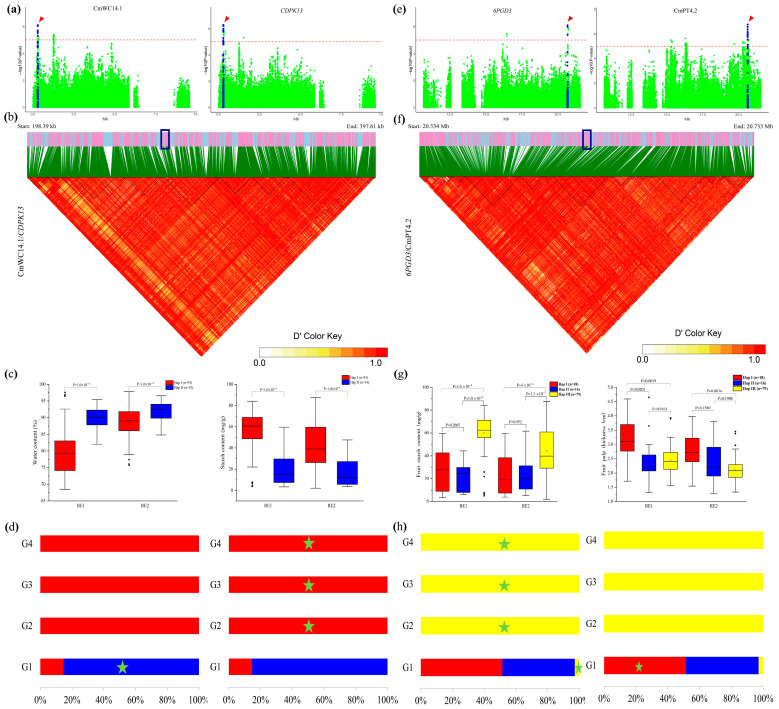
The repeatedly identified signals of selective sweeps and GWAS of QTL-colocalization. (**a**,**e**): Local Manhattan plot of *CmWC14.1*/*CDPK13* and *6PGD3*/*CmPT4.2*; (**b**,**f**): local LD block analysis of *CmWC14.1*/*CDPK13* and *6PGD3*/*CmPT4.2*; (**c**,**g**): haplotype analysis of *CmWC14.1*/*CDPK13* and *6PGD3*/*CmPT4.2*; (**d**,**h**): the distribution in different pumpkin subpopulations for *CmWC14.1*/*CDPK13* and *6PGD3*/*CmPT4.2*. Putative genes associated with each signal are highlighted in blue boxes within the LD heatmaps. For haplotype analysis, the “n” values in histograms and boxplots represent the number of accessions carrying the corresponding haplotypes. In boxplots, the lower and upper edges of boxes indicate the 25% and 75% quartiles, respectively; central lines denote medians, and small hollow squares mark means. Whiskers extend to 1.5× the interquartile range, with small solid diamonds indicating outliers. *p*-values from two-sided Student’s *t*-tests are displayed above the boxplots. The olive-green star indicates the haplotypes related to a higher water content, starch content, starch content and pulp thickness, respectively (from (**left**) to (**right**)).

In total, 8 signals associated with STA, CL, PT and WC were further analyzed to identify the putative genes (Figure 6 and Figure 7 and Supplementary Figure S2). Among them, *CmCL1.1* had two candidate genes (*Cmax01G001700* and *Cmax01G001705*) (Supplementary Figure S2a,b). *CmCL1.1-1* namely *Cmax01G001700* encodes a WRKY transcription factor with four haplotypes (Supplementary Figure S2c). Three haplotypes, Hap I, Hap II and Hap III had no remarkable differences in cellulose content. Hap IV (4.88 mg/g), which consists entirely of G1 accessions, presented higher CL than the other three haplotypes in replication 1 (RE1) while the four haplotypes had no significant difference in RE2 (Supplementary Table S7). The gene encoding an ethylene-responsive transcription factor (*CmCL1.1-2*, *Cmax01G001705*) was also considered as the putative gene for the CL signal. Hap I, which comprises the G1 population, accounted for 32.3% of the mutation type of *CmCL1.1-2* and presented the highest CL in both RE1 and RE2 (Supplementary Figure S2c,d). The *CmCL1.1-2* is homologous to *ERF IIId* genes (including *AtERF34*, *AtERF35*, *AtERF38* and *AtERF39*) that regulate the biosynthesis of cell walls [25]. Two haplotypes of *CmPT11.1* (*Cmax11G000862*) were verified in 83 accessions, with Hap II accessions belonging to G1 having a thicker pulp thickness (PT) in both RE1 and RE2 (Figure 6c,d). This gene encodes an isopentenyl diphosphate delta-isomerase (IPPD), which catalyzes a crucial activation step in the biosynthesis of isoprenoids, including cytokinin and gibberellin biosynthesis pathway [26]. *CmCL5.1* contained a glucan endo-1,3-beta-glucosidase-encoding gene *Cmax05G001141*, which contributes to cellulose breakdown by acting on cellobiose and other small cello-oligosaccharides produced during cellulose hydrolysis by other enzymes like cellulases [27]. A total of 95 accessions were classified into three haplotypes and Hap I showed the lowest cellulose content in RE1 indicating that *C. maxima* had relatively higher cellulose content than *C. moschata* (Figure 6c,d). *CmWC14.1* with the gene *CDPK13*, contains a protein kinase domain followed by a unique calmodulin-like domain with four calcium binding sites (Figure 7a,b) [28]. The overexpression of its orthologous gene *OsCDPK1* has been found to regulate starch accumulation in rice [29]. A total of 93 Hap I accessions had more starch but less water content, indicating that starch content and water content exhibited a negative correlation in pumpkin fruit (Figure 7c). The 85.36% if the G1 subgroup contained low starch while its moisture content was high (Figure 7d). Similarly, *CmPT4.2* overlapped with the gene *6PGD3*, which encodes 6-phosphogluconate dehydrogenase which is critical for endosperm starch accumulation in maize (Figure 7e,f) [30]. Three haplotypes were detected in the 113 accessions, and Hap III accessions showed significantly higher starch content in the two distinct locations (Figure 7g). The accessions with high starch content predominantly existed in the G2, G3 and G4 populations. However, la arger PT (3.23 cm) accounting for 50% of the G1 population was observed with Hap I in RE1 not in RE2 (Figure 7g,h, Supplementary Table S7).

### 2.6. Yield and Plant Growth Variations

**Figure 8:**
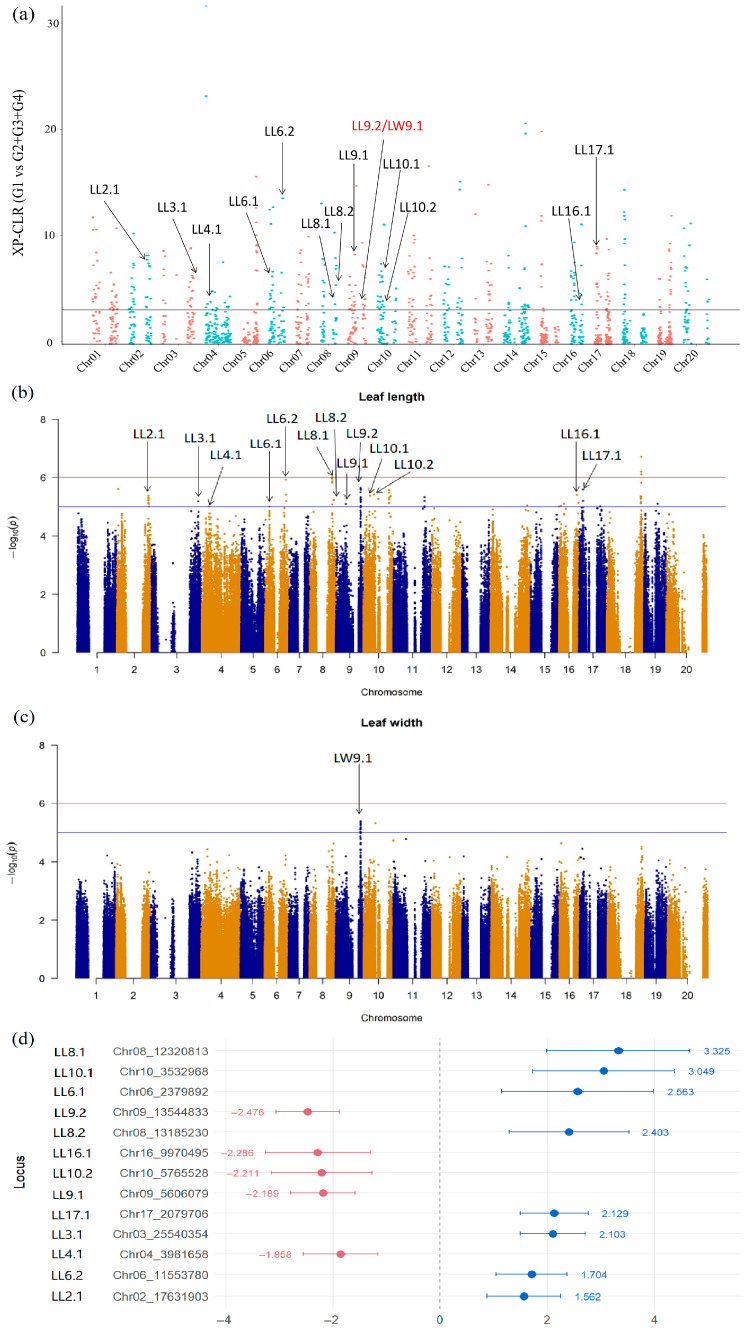
Leaf length and leaf width related genes mining in pumpkin. (**a**): Genome-wide distribution of selective sweeps through the comparison of G1 vs G2 + G3 + G4; (**b**,**c**): Manhattan plot of GWAS analysis for LL and LW; (**d**) Forest plot of allele substitution effects for 13 Loci. Overlapped gene in the selective regions and GWAS are indicated in red text. In forest plot, the left and right error lines represent the 95% confidence interval, red and blue dots indicate negative and positive allele substitution effect, respectively.

The yield of pumpkin is significantly associated with plant phenotypic factors such as leaf length (LL), and LW. From the perspective of pumpkin diversification, *C. maxima* exhibited an obviously shorter LL than *C. moschata*. Within the smaller subgroups, the G2 subpopulation showed the shortest LL (Supplementary Figure S3a). On the other hand, no statistically significant difference was observed in LW between *C. maxima* and *C. moschata*. Similarly, the G2 subpopulation exhibited the smallest LW compared to the other three subpopulations, as well (Supplementary Figure S3b). In total, 13 signals associated with LL were identified in both the GWAS and XP-CLR analyses, while only one signal associated with LW was detected (Figure 8a–c, Supplementary Table S6). This overlapping signal (LL9.2/LW9.1) located on chromosome 9 was repeatedly identified in the LL and LW GWAS analyses, indicating a close association of this signal with leaf area. The allele substitution effects (ASE) of each signal were exhibited in Figure 8d. Three ASE values of QTLs were higher than that of LL9.2, indicating these four QTLs might play more important roles in the trait of LL. QTLs including LL8.1, LL10.1 and LL6.1 had a positive effect on LL while LL9.2 had a negative effect on LL (Figure 8d). A LOB domain-containing protein (LBD) encoded by the gene *Cmax08G000859* was identified in the LL8.1 locus. Although LBD family genes are well known to regulate leaf size, shape, and adaxial-abaxial polarity during leaf morphogenesis in *Arabidopsis thaliana*, *Oryza sativa* and *Medicago truncatula* [31], we detected no statistically significant difference in LL between the Hap I and Hap II haplotypes of *Cmax08G000859*, indicating that *Cmax08G000859* is unlikely to be the causal candidate gene for LL regulation (Supplementary Figure S4a). In the QTL of *LL10.1*, SNP variation analysis revealed a total of 3 nonsynonymous mutations in the gene of *CmLL1* (*Cmax10G000699*), which is a member of TIFY protein family. The overexpression of the homologous gene *OsTIFY11b* promotes leaf elongation potentially by increasing cell division [32]. Three haplotypes of *CmLL1* were observed, and *CmLL1*-Hap I exhibited the shortest LL (Supplementary Figure S4b). In the QTL of LL6.1, we identified a gene *CmLL2* (*Cmax06G000433*) encoding an OVATE family protein, which has been found to regulate cell elongation and participate in the shape of various organs including hypocotyls, leaves, stems in rice and *Arabidopsis* [33,34,35]. *CmLL2*-Hap II had an average LL of 25.98 cm in RE1 and 32.03 cm in RE2, which was significantly longer than that of *CmLL2*-Hap I (Supplementary Figure S4c, Supplementary Table S7).

**Figure 9:**
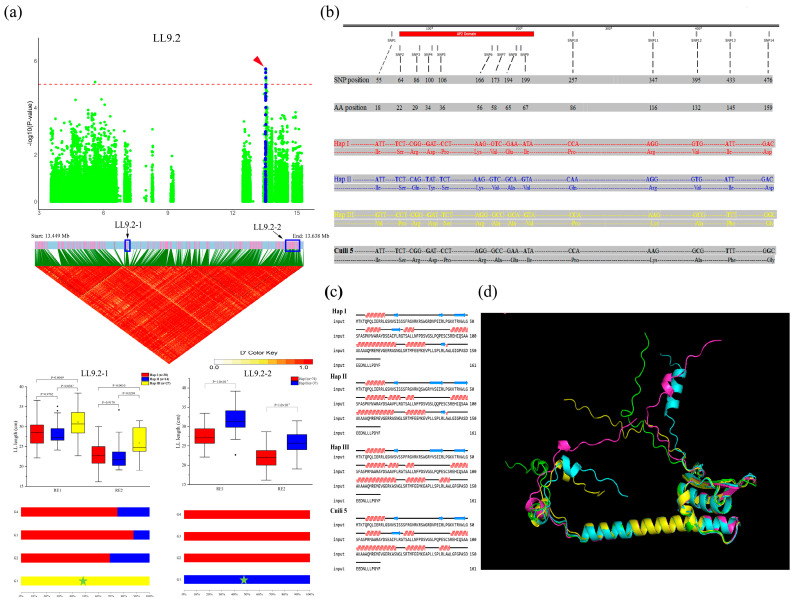
The candidate gene analysis for QTL LL9.2. (**a**): Manhattan plots (**top**), LD block analysis (**upper-middle**), haplotype analysis (**lower-middle**) and the distribution in different pumpkin subpopulations (**bottom**); (**b**): the distribution of 14 nonsynonymous mutations in the gene Cmax09G001045 of 4 haplotypes; (**c**,**d**): the secondary and three-dimensional structures of proteins encoded by Cmax09G001045 of 4 haplotypes. The red triangle indicates the identified SNPs associated with LL phenotype in the Manhattan plot. Putative genes associated with each signal are highlighted in blue boxes within the LD heatmaps. For haplotype analysis, the “n” values in histograms and boxplots represent the number of accessions carrying the corresponding haplotypes. In boxplots, the lower and upper edges of boxes indicate the 25% and 75% quartiles, respectively; central lines denote medians, and small hollow squares mark means. Whiskers extend to 1.5× the interquartile range, with small solid diamonds indicating outliers. *p*-values from two-sided Student’s *t*-tests are displayed above the boxplots. The olive-green star indicates the haplotypes related to a longer leaf length. In protein 3D structure, Hap I, Hap II, Hap III and ‘Cuili 5” are showed in cyan, yellow, pink and green, respectively.

For the overlapping QTL LL9.2 or LW9.1, two candidate genes (*Cmax09G001045* and *Cmax09G001050*) were regarded as being responsible for leaf area in the LL9.2/LW9.1 locus. SNP variation analysis revealed 14 nonsynonymous mutations in the exonic zone of *Cmax09G001045*, which primarily encodes an ERF, and three haplotypes were identified (Figure 9a). A previous study demonstrated that overexpression of the Chinese cabbage gene *BrERF4* in *Arabidopsis thaliana* plants resulted in reduced leaf size through the inhibition of cell expansion [36]. Therefore, *CmLL3* (*Cmax09G001045*) may modulate leaf length by indirectly regulating the transcription of ethylene-responsive genes to induce plant growth retardation. No big difference between Hap I and Hap II was found while Hap III likely contributes to a longer LL (Figure 9a). Similarly, *Cmax09G001050* annotated as a glycerophosphodiester phosphodiesterase participates in phosphate homeostasis and has been found to reduce shoot length in the *OsGDPD13* mutant [37]. Two haplotypes were observed and Hap II distinctly exhibited a longer LL than Hap I in the two replicates (Figure 9a). All accessions with a longer LL belonged to the G1 subpopulation in the QTLs LL9.2-1 and LL9.2-2. In addition, G1 and G2 shared the lowest LW among the four subpopulations (Supplementary Figure S3b). However, the distinct haplotypes of the two putative genes did not show substantial LW differences, possibly because of the insufficient number of accessions in subgroups G3 and G4.

‘Cuili 5’, our newly developed high-quality hybrid variety of *C. maxima*, exhibits a leaf length of 25 cm and a leaf width of 35 cm. In actual production and cultivation, its characteristic of exceptionally small leaves ensures good field ventilation and light transmission, leading to significantly reduced the severity of plant diseases and robust plant growth. To investigate the mechanism underlying its small leaf trait, we performed resequencing and sequence alignment for this variety, and the results revealed a novel haplotype in the gene *Cmax09G001045,* while no detected variations existed in *Cmax09G001050*. As no intron was detected in the gene *Cmax09G001045*, this gene only has one exon and a typical ERF conserved domain AP2. Eight SNPs located in the AP2 domain exhibited diverse variations in different haplotypes. ‘Cuili 5’ and Hap III share consistent variations at 9 SNPs (3, 4, 6, 7, 10, 11, 12, 13, 14), while Hap II and Hap III exhibit consistent variations at 3 SNPs (5, 8, 9) (Figure 9b). This suggests that the small leaf phenotype is most likely dominated by two variations, namely SNP1 and SNP2. Regarding the secondary and tertiary structures of the protein, the predicted results indicated that the four haplotypes maintain relative stability in their basic α-helix and β-sheet architectures (Figure 9c,d). Hap I and Cuili 5 exhibited similar secondary structures in the first 50 amino acids but differed in their three-dimensional spatial conformations. Similarly, Hap I and Hap III shared comparable secondary structures in the 100–150 amino acid region, yet there were differences in their three-dimensional spatial configurations, as well (Figure 9c,d). The results also confirmed that the sequence within the first 50 amino acids of this gene, which contains the anterior part of the AP2 domain, is more critical.

### 2.7. Candidate Gene Expression Analysis During Fruit Development

**Figure 10:**
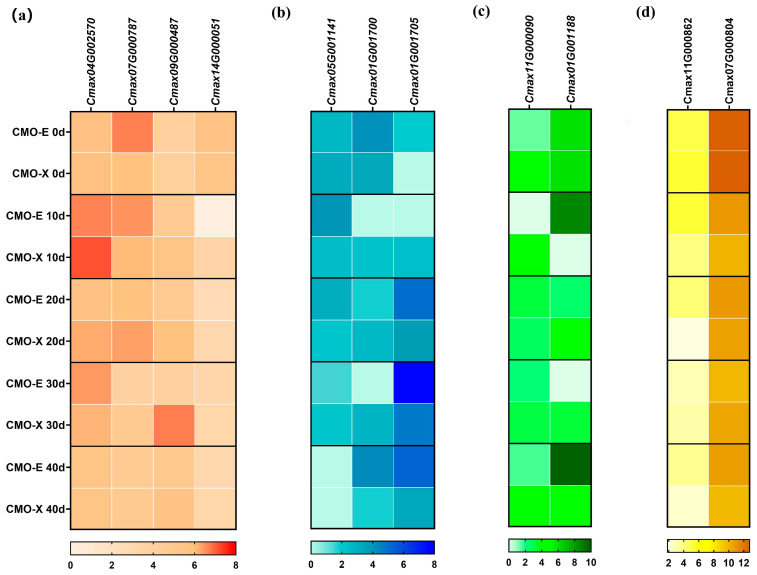
Heatmaps of candidate gene expressions during different developmental stages of *C. moschata* fruit. (**a**) Genes involved in starch metabolism; (**b**) genes involved in cellulose metabolism; (**c**) genes involved in fruit color; (**d**) genes involved in pulp thickness and water content. Color scheme was based on a log_2_(FPKM + 1) transformation.

To verify the gene expressions of candidate genes related to fruit development, RNA-Seq data (SRP259432) from a previous study [11] were utilized and analyzed in Figure 10. In general, a total of 11 identified candidate genes were detected in two *C. moschata* varieties (CMO-E and CMO-X) from 0 d, 10 d, 20 d, 30 d, and 40 d of fruit development. Four genes (*Cmax04G002570*, *Cmax07G000787*, *Cmax09G000487*, *Cmax14G000051*) involved in starch metabolism showed different expression patterns (Figure 10a). The expression level of the gene *6PGD3* (*Cmax04G002570*) increased rapidly and reached its peak at 10 days after fruit set, and its expression level in the CMO-X line was significantly higher than that in the CMO-E line. The expression of gene *FBA6* (*Cmax07G000787*) in CMO-E decreased progressively with increasing days after fruit set, whereas in CMO-X, it exhibited a trend of initial increase followed by a decrease, with its expression level reaching the peak at 20 d. The expression of *ISA3* (*Cmax09G000487*) remained stable throughout fruit development in CMO-E, while it reached a high expression level (log_2_(FPKM + 1) > 6) at 30 d in CMO-X. The expression of gene *CDPK13* (*Cmax14G000051*) remained stable throughout fruit development, with no significant difference between CMO-E and CMO-X, suggesting that this gene is unlikely to participate in starch biosynthesis.

Three candidate genes (*Cmax05G001141*, *Cmax01G001700*, *Cmax01G001705*) were deduced to participate in cellulose synthesis (Figure 10b). The expression level of the gene *CmCL5.1* (*Cmax05G001141*) was maintained at a moderate level in both the CMO-E and CMO-X lines at 0 d, and showed an overall downward trend with the extension of fruit development time, reaching the lowest expression level at 40 d, with a higher expression level in CMO-E than that in CMO-X both at 10 d and 20 d. At 0 and 40 days after fruit set, the expression level of the gene *CmCL1.1-1* (*Cmax01G001700*) in CMO-E was significantly higher than that in CMO-X. The expression of *CmCL1.1-2* (*Cmax01G001705*) in CMO-E was significantly higher than that in CMO-X at 20 d, 30 d and 40 d.

The expression of the gene *KNAT6* (*Cmax11G000090*) remained stable at a moderate level in both CMO-E and the CMO-X lines during the whole fruit development process, although a decrease was observed in CMO-E at 10 d, and there was no significant difference between the two lines (Figure 10c). The gene *ERF4* (*Cmax01G001188*) was active in both lines at 0 d. At 10 d, its expression dropped significantly in CMO-E, while it rose sharply in CMO-X (log_2_(FPKM + 1) > 8). During 20 d to 30 d, the expression of this gene in both lines fell back to a moderate level. At 40 d, the expression increased again in both lines, and the expression level in CMO-E was much higher than that in CMO-X. The last two candidate genes (*Cmax11G000862*, *Cmax07G000804*) also exhibited different expression characteristics during fruit development (Figure 10d). The expression of *Cmax11G000862* showed a descending trend during fruit development, with lower expression in CMO-X than in CMO-E. The expression pattern of the gene *Cmax07G000804* was similar between the two lines. It decreased after 0 d and subsequently tended to stabilize, yet its expression level remained consistently higher in CMO-E.

## 3. Discussion

Pumpkin (*Cucurbita* spp.), is a globally vital crop with significant agricultural, economic, and nutritional value, serving as an important source of carbohydrates, vitamins, and antioxidants in diverse diets [38,39]. Its extensive phenotypic diversity—encompassing traits such as fruit size, shape, and nutritional composition, as well as leaf morphology and stress tolerance—makes it an excellent model system for studying plant domestication, trait evolution, and adaptive diversification. However, despite its agricultural importance, the genetic mechanisms underlying many key agronomic traits remain poorly characterized. This knowledge gap hinders progress in molecular breeding efforts aimed at enhancing yield, quality, and resilience. Addressing this gap is crucial for accelerating the development of improved pumpkin varieties tailored to changing agricultural environments and market demands. Here, we re-sequenced 146 accessions of pumpkins consisting of 51 *C. moschata* and 95 *C. maxima* germplasms, and constructed a pumpkin genomic variation map which provided abundant genetic variants, serving as a robust tools to elevate both fundamental research and breeding applications in pumpkin. Compared to other Cucurbitaceae species, the divergence event between *C. maxima* and *C. moschata* emerged much later, approximately 3 million years ago [8]. Then they developed different adaptive strategies during domestication that *C. moschata* exhibits stronger stress resistance in natural environments, while *C. maxima* is likely to contain higher starch and sugar, and lower β-carotene, depending on artificial selection [40,41,42]. Our results also demonstrated that the G1 subpopulation namely *C. moschata* had the lowest starch and amylose content, but the highest soluble sugar and β-carotene among the four subgroups (Supplementary Figure S3c–f).

Meteorological conditions are critical environmental drivers of pumpkin phenotypic expression, and their interaction with field management contributed to the observed trait variation between the two experimental sites. The above-average temperatures in the seedling stage accelerated seedling growth, while the continuous rainy and low-light events during flowering and fruit setting caused excessive vegetative growth, increased the fruit malformation rate, and led to a wider variation in fruit shape. For fruit quality traits, the relatively small diurnal temperature difference and excessive precipitation during fruit expansion slightly reduced the accumulation of soluble sugars and starch, while insufficient sunshine significantly inhibited β-carotene biosynthesis. Overall, the meteorological conditions during the 2024 spring growing season were within the suitable range for pumpkin cultivation in the Yangtze River Delta, and our two-site experimental design effectively mitigated the impact of micro-environmental differences, ensuring the reliability of the phenotypic identification results.

Fruit quality comprises appearance quality and intrinsic quality, which are strongly influenced by human selection [43]. The exploration of fruit quality traits in pumpkin through GWAS has proven to be informative. In our study, the identification of multiple loci associated with traits such as rind color, pulp color, PT, and STA is consistent with previous studies in related crops, highlighting the conserved genetic mechanisms governing these traits. For instance, the discovery of *CmaKNAT6* in the rind color-associated QTL on chromosome 11 is consistent with the known role of knotted1-like homeobox (KNOX) genes in regulating chlorophyll accumulation and chloroplast development in tomato and watermelon [44,45]. The presence of the ethylene-responsive transcription factor 4 (ERF4) gene in the pulp color-associated region further supports the established roles of *SlERF6* and *MdERF3* in modulating fruit ripening and carotenoid/anthocyanin accumulation [46,47]. These results not only validate the power of GWAS in uncovering the genetic basis of complex traits in pumpkin but also provide valuable candidate genes for further functional validation. The convergent evolution signals detected in traits like STA, PT, and WC suggest that both natural and artificial selection have played crucial roles in shaping these traits. The G1 subpopulation showed lower STA but higher WC values than the G2, G3 and G4 subpopulations, which might be attributed to its favorable haplotype of the gene *CmSAT1* (*Cmax14G000051*) (Supplementary Figure S3c, Supplementary Tables S4 and S7). The *Arabidopsis thaliana CDPK4/5/6/11* interacted with the hydrotropism-specific protein MIZU-KUSSEI1 to regulate the hydrotropic growth of plants [48]. Meanwhile, CDPKs were found to be involved in regulating sugar levels in the fruit pulp, which could influence water content [49]. This is in line with the general understanding that domestication and human-driven selection often lead to the convergence of beneficial traits across different populations [50].

To verify the functions of these candidate genes, we re-analyzed the published time-course transcriptome dataset (SRP259432) to validate the expression patterns of 11 candidate genes related to fruit development and quality formation across 0–40 d after fruit set in two germplasms (CMO-X and CMO-E) with distinct fruit quality, revealing the association between gene expression divergence and phenotypic variation between the two lines [11]. For the four starch metabolism-related candidate genes, *6PGD3* (*Cmax04G002570*), *FBA6* (*Cmax07G000787*) and *ISA3* (*Cmax09G000487*) showed significant stage-specific high expression in the high-quality germplasm CMO-X, which was highly consistent with the higher dry matter and starch accumulation, as well as the superior flesh texture of CMO-X (Figure 10a). Whereas *CDPK13* (*Cmax14G000051*) exhibited no significant expression difference between the two lines throughout fruit development; thus it was excluded from the key regulators for starch biosynthesis divergence in pumpkin. Three cellulose synthesis-related genes (*Cmax05G001141*, *Cmax01G001700*, *Cmax01G001705*) had significantly higher expression levels in CMO-E at multiple developmental stages (Figure 10b). This expression pattern perfectly matched the fibrous and rough flesh texture of CMO-E, indicating these genes are critical candidates regulating the fibrous mouthfeel of pumpkin fruit. In addition, the pulp thickness-related gene *Cmax11G000862* and water content-related gene *Cmax07G000804* showed consistently higher expression in CMO-E, which was in line with the higher water content and lower dry matter phenotype of CMO-E (Figure 10d). The transcription factor *KNAT6* (*Cmax11G000090*) had no significant expression difference between the two lines, while *ERF4* (*Cmax01G001188*) presented significant genotype- and stage-specific expression patterns, suggesting its stage-specific regulatory role in pumpkin fruit development and ripening (Figure 10c). Collectively, this study validated key candidate genes regulating core fruit quality traits including starch accumulation, fiber texture and water content in pumpkin.

The investigation into the genetic basis of LL in pumpkin also yielded interesting results. The identification of multiple signals associated with LL, especially the overlapping signal (LL9.2/LW9.1) related to leaf area, provides potential targets for breeding for an optimal leaf size. Generally, *C. moschata* has a longer LL than *C. maxima* and the G2 subpopulation has the shortest LL among the four subgroups (Supplementary Figure S3a). The candidate genes identified, such as *Cmax09G001045* encoding an ERF, are is implicated in plant leaf development. A total of 14 favorable alleles for LL were identified, and *CmLL2*-Hap III exhibited longer LL than *C. maxima* (Figure 9). Intriguingly, the discovery of a novel haplotype in *CmLL3* in the ‘Cuili 5’ hybrid variety, which is associated with its small leaf trait, offers a unique opportunity to understand the molecular mechanism underlying leaf size variation. This finding is significant as leaf size is closely related to the canopy microclimate [51]. Smaller leaves allow more light to penetrate the canopy, improving photosynthesis in lower leaves. In crops like soybean, upright leaves with reduced chlorophyll in upper canopies enhance light distribution and reduce self-shading. Conversely, large leaves in dense canopies lead to light saturation in the upper layers and light limitation below, affecting overall productivity [52,53,54]. By understanding the genetic basis of leaf size, breeders can potentially develop pumpkin varieties with improved agronomic performance, such as better light interception and reduced disease incidence, as demonstrated by the characteristics of ‘Cuili 5’ in actual production.

Collectively, our results clarify the genetic basis of key agronomic traits in pumpkin, reveal the domestication and selection history of *C. maxima* and *C. moschata*, and provide abundant genomic resources and molecular markers for marker-assisted breeding. Future research will focus on three main directions: (1) functional validation of the key candidate genes identified in this study using CRISPR/Cas9-mediated gene editing and overexpression experiments; (2) systematic histochemical and transcriptomic analyses of fruit cuticles to elucidate the molecular mechanisms of pumpkin fruit storage durability; and (3) investigation of the genetic architecture of root traits (for example, starch content) and abiotic stress tolerance, and their correlation with fruit quality and yield. These studies will further deepen our understanding of pumpkin biology and facilitate the development of superior varieties through molecular breeding.

## 4. Materials and Methods

### 4.1. Plant Materials and DNA Isolation

A total of 146 pumpkin accessions were collected and obtained from the Shaoxing Academy of Agricultural Sciences for re-sequencing. This collection comprised 51 *C. moschata* and 95 *C. maxima*. High-quality DNA samples were extracted from the 146 pumpkin germplasm resources using the E.Z.N.A. Tissue DNA kit (Omega Bio-Tek, Norcross, GA, USA) following the Tissue DNA Spin Protocol [55]. Post-extraction, quality control assessments were performed, and only qualified DNA samples (≥3 µg in quantity, ≥30 ng/µL in concentration, and an OD260/OD280 ratio of 1.80–2.00) were selected for subsequent analyses.

### 4.2. Library Preparation and Re-Sequencing

For each sample, at least 3 μg of genomic DNA was used to construct paired-end libraries (average insert size ~450 bp) following Illumina’s standard protocols. DNA was fragmented using a Covaris, and then subjected to end repair, A-tailing, and adapter ligation. Fragments were purified by gel electrophoresis, polymerase chain reaction (PCR)-amplified, and indexed for multiplexing. Sequencing was performed on the Illumina NovaSeq 6000 platform at Shanghai Biozeron Biotechnology Co., Ltd. (Shanghai, China) [56].

### 4.3. Reads Processing and Single Nucleotide Polymorphism (SNP) Calling

Raw reads were filtered to remove adapter sequences and low-quality reads (with >10% N and >50% Q10) to produce clean reads. For each accession, all clean reads were aligned to the reference *C. maxima* (HZAU) genome using the MEM algorithm in the Burrows–Wheeler Aligner (BWA) software v0.7.19 (bwa-mem -k 32) [57]. SAM format files were sorted and merged using SAMtools v1.23, and duplicate reads were removed with Picard (v1.92) [58]. Custom Perl v5.30 scripts were used to calculate the sequencing depth and coverage from the alignments. Valid BAM files were processed with the GATK (v4.1.2.0) “Haplotype Caller” to detect SNPs and short insertions and deletions (InDels) [59]. The resulting variants were quality-filtered using the VCFtools’ Variant Filtration module with the following parameters: QD < 2.0, FS > 60.0, MQ < 40.0, or SOR > 10.0. The filtered variants were then annotated with ANNOVAR v20250721 to classify SNPs as synonymous or non-synonymous and to categorize InDels [60]. Structure variations (SV) were identified using BreakDancer v1.1_20100719 [61]. Circos v.0.6930 was used to create a genomic representation of the genetic indices [62].

### 4.4. Population Structure Analysis

First, genetic distances were computed using GCTA v1.93.2 with the SNP data, followed by the construction of a neighbor-joining (NJ) tree via FastTree v2.1.10 to infer phylogenetic relationships [63,64]. Principal component analysis (PCA) was performed on the pumpkin population using GCTA v1.93.2 to decompose the genetic matrix into eigenvectors, and the resulting eigenvectors were visualized to reveal population clustering patterns. PLINK v1.9 was used to generate input files, which were then analyzed with Admixture v1.3.0—a maximum likelihood-based program—with the number of assumed ancestral populations (K) set from 2 to 9 [65,66]. To assess linkage disequilibrium (LD), Haploview 16 was used to calculate allele correlation coefficients (r^2^) in the different populations [67].

### 4.5. Genomic Variation

Genome-wide estimates of average pairwise divergence (θπ) and Watterson’s estimator (θw) were computed for the compared populations. θπ, θw, and Tajima’s D were calculated across the genome using sliding windows with a 50% overlap between adjacent windows, and these parameters were computed for each window via an in-house PERL script. A 100 kb window size was used to visualize genomic patterns, and population differentiation was quantified using FST.

### 4.6. Field Experiments and Phenotype Measurement

In the spring of 2024, all pumpkin accessions were planted in duplicate trials at two sites located in Shaoxing with distinct management regimes: the Wuhe Research Base of the Shaoxing Academy of Agricultural Sciences (RE1; 30°02′ N, 120°70′ E), managed by research institutions, and the site in Dongjianhu Future Farm (RE2; 29°99′ N, 120°74′ E) operated under the management of production entities. Meteorological data covering the entire pumpkin growth period were obtained from the official data of Shaoxing Meteorological Bureau. During the experimental period, the average temperature was 20.0 °C, which was 1.1 °C higher than the historical average. Specifically, two continuous rainy and low-light events (over 3 consecutive days) occurred during the flowering and fruit-setting period (late April to early May), and 4 days of high temperatures with daily maximum temperature ≥ 35 °C occurred during the fruit expansion period (mid-May to early June). The two experimental sites shared the same regional macro-meteorological background, with slight differences in the field micro-environment. The fruit shape, rind color and flesh color were correspondingly assigned values according to different classifications for analysis. Other growth parameters like leaf length and width were measured based on at least five replications. The fruit quality indicators including starch, amylose, soluble sugar, cellulose, β-carotene and water content were assessed using relevant quality analysis kits following the manufacturers’ instructions (Hanwu Ji, Hangzhou, China). All fruit samples used for quality analysis were collected from the mesocarp tissue, which is the main edible part of the pumpkin. As confirmed by previous cytological studies [68], the pumpkin pericarp (rind) develops from the ovary wall and consists entirely of diploid (2n = 40) somatic cells, consistent with the ploidy level of the mesocarp cells. Therefore, ploidy differences do not affect the accuracy of our phenotypic measurements or genetic association analyses.

### 4.7. GWAS and Haplotype Analysis

Association analyses were conducted using general linear models (GLMs) and mixed linear models (MLMs) with TASSEL v.2.1 [69]. A kinship matrix (K-matrix), representing the pairwise relationship matrix calculated by TASSEL v.2.1, and the population structure (Q-matrix) calculated by Admixture as a correction for population structure, were integrated in the MLM association models to calculate *p*-values for associating each SNP marker with the trait of interest ad avoiding spurious associations by TASSEL v.2.1. Results were compared to determine the best model for our analysis. Considering that the genome-wide average LD decay distance in *C. maxima* (at r^2^ = 0.50) is 106 kb, adjacent GWAS loci within this 106 kb range were grouped as a single GWAS interval or signal.

All genes within the GWAS signal interval or 106 kb flanking regions were extracted as putative candidates. LDblockShow (v.1.41) was then employed to visualize local LD patterns in these regions [70]. For each GWAS signal, SNPs within LD blocks were ranked by ascending *p*-value (<10^−5^), prioritizing variants with the strongest statistical associations. Genes overlapping or in proximity to these top-ranked variants were flagged as primary candidates. Functional annotation and homology evidence were integrated to prioritize genes with plausible biological roles. Concurrently, haplotype analysis of the candidate genes was performed to assess population-level correlations between haplotypic variation and phenotypic traits, thereby validating their genetic effects.

### 4.8. Detection of Selective Sweeps

Selective sweeps were performed using the cross-population composite likelihood ratio test by cross-population composite likelihood ratio test (XP-CLR) package (v.1.0) [71]. The sliding window and step size were set as 100 kb and 10 kb, respectively. Differentiation sweeps were identified by comparing *C. moschata* and *C. maxima*, while improvement sweeps were detected by pairwise comparisons among the G2, G3 and G4 subgroups. Genomic regions in the top 5% of the XP-CLR scores were prioritized as candidate selective sweeps, and genes within these regions were annotated as potential targets of selection.

### 4.9. Transcriptome Analysis

The transcriptional profiles of candidate genes from the low-quality variety CMO-E and the high-quality variety CMO-X genotypes were downloaded under accession number SRP259432 [11]. The expression levels of 11 candidate genes were quantified using the fragments per kilobase of transcript per million mapped fragments (FPKM) method across five critical fruit developmental stages (0 d to 40 d after fruit set).

### 4.10. The Prediction of Protein Structure

The conserved domains of the candidate gene sequence were analyzed using the online software MOTIF Search (https://www.genome.jp/tools/motif/, accessed on 3 July 2025). The secondary structure of the candidate protein sequences was analyzed via SOPMA (https://npsa-prabi.ibcp.fr/cgi-bin/npsa_automat.pl?page=/NPSA/npsa_sopma.html, accessed on 3 July 2025), and their tertiary structures were further constructed using trRosetta (https://yanglab.qd.sdu.edu.cn/trRosetta/, accessed on 3 July 2025). Using PyMOL software v3.1, the predicted 3D structures of varied proteins were visualized.

### 4.11. Statistical Analysis

All statistical analyses were performed using the R software (v4.3.1) and OriginPro (v2024b, OriginLab Corporation, Northampton, MA, USA). Descriptive statistics including the mean, standard deviation (SD), median, and quartiles were calculated for all phenotypic traits. For pairwise comparisons between two groups, a two-tailed Student’s *t*-test was used. For multiple comparisons among three or more groups, one-way analysis of variance (ANOVA) followed by Duncan’s multiple range test (*p* < 0.05) was performed.

For the GWAS analysis, the significance threshold was set at *p* < 1 × 10^−5^ after Bonferroni correction for multiple testing. The quantile–quantile (Q-Q) plots were generated to evaluate the performance of the association models and assess the inflation of *p*-values due to population structure. For haplotype analysis, the differences in phenotypic values between different haplotypes were compared using two-sided Student’s *t*-tests. The allele substitution effects (ASE) of significant GWAS loci were estimated using a linear regression model, with the phenotypic trait as the dependent variable and the genotype (coded as 0, 1, 2) as the independent variable.

All statistical tests were two-sided, and *p*-values less than 0.05 were considered statistically significant unless otherwise specified. Detailed statistical parameters and results are provided in the figure legends and Supplementary Tables.

## 5. Conclusions

This study presents a comprehensive population genomic analysis of 146 accessions representing the two most economically important pumpkin species *C. maxima* and *C. moschata*, and underscores the genome-wide genetic underpinnings of their important agronomic traits. Employing an integrated approach that encompasses population structure analysis, GWAS, selective sweep detection, and the identification and validation of candidate genes, we have substantially deepened our understanding of genetic diversity patterns, population divergence mechanisms, and the genetic basis of phenotypic variation in pumpkin. The results provide valuable resources and markers for marker-assisted breeding, aiding in the improvement of productivity, quality, and adaptability to meet global agricultural and nutritional needs. Future research will focus on the functional validation of key candidate genes, the dissection of the regulatory networks governing fruit quality formation, and the exploration of genetic loci associated with abiotic stress tolerance and root traits.

## Figures and Tables

**Table 1:** Genomic location of SNPs.

Type	Number of Variants	Percentage (%)
exonic	1,278,300	12.19
intergenic	3,991,796	38.05
intronic	2,771,565	26.42
splicing	6074	0.06
upstream	1,175,481	11.21
downstream	1,057,420	10.08
upstream;downstream	209,250	1.99

**Table 2:** Genomic location of InDels.

Type	Number of Variants	Percentage (%)
exonic	27,561	1.39
intergenic	860,798	43.43
intronic	486,771	24.56
splicing	1288	0.06
upstream	290,028	14.63
downstream	259,258	13.08
upstream;downstream	56,334	2.84
UTR5	58	0.00

**Table 3:** Polymorphism information content (PIC), expected heterozygosity (He), average observed heterozygosity (Ho) and inbreeding coefficient (FIS) calculated for the entire population and for each subpopulation.

Group	PIC	He	Ho	Fis	I (Shannon’s Information Index)	Tajima’s D
All accessions	0.313	0.396	0.023	0.910	0.622	0.235
G1 (*C. moschata*)	0.072	0.089	0.055	0.583	0.159	−0.108
G2 (*C. maxima* green-skinned)	0.036	0.043	0.008	0.741	0.088	−0.143
G3 (*C. maxima* gray-skinned)	0.031	0.038	0.006	0.748	0.070	−0.218
G4 (*C. maxima* red-skinned)	0.039	0.049	0.009	0.767	0.093	−0.194

## Data Availability

All of the raw reads of the pumpkin accessions generated in this study have been deposited in the public database of National Center of Biotechnology Information under PRJNA1293787.

## Supplementary Materials

The following supporting information can be downloaded at: https://www.mdpi.com/article/10.3390/ijms27114903/s1.

## Abbreviations

The following abbreviations are used in this manuscript:

AMY	amylose content
ASE	allele substitution effects
βCAR	β-carotene content
BND/CTX	Interchromosomal translocations
CL	cellulose content
CV	Coefficient of Variation
D	genetic distance
DEL	Deletions
ERF	Ethylene Responsive Factor
FIS	Inbreeding Coefficient
FPKM	Fragments Per Kilobase Of Transcript Per Million Mapped Fragments
FST	Fixation Index
GLM	General Linear Model
GLMQ	GLM with Q-matrix
GWAS	Genome-Wide Association Study
He	Heterozygosity
Ho	Heterozygosity
I	Shannon’s information index
InDels	Insertions And Deletions
INS	Insertion-Type Structural Variation
INV	Inversion-Type Structural Variation
IPPD	isopentenyl diphosphate delta-isomerase
KNOX	knotted1-like homeobox
LBD	LOB domain-containing protein
LD	Linkage Disequilibrium
LL	Leaf length
LW	leaf width
MLM	Mixed Linear Model
MLMk	MLM with K-matrix
MLMkq	MLM with both K and Q matrices
PCA	Principal Component Analysis
PCR	Polymerase Chain Reaction
PIC	Polymorphism Information Content
PT	pulp thickness
QTL	Quantitative Trait Loci
RE	Replication
RILs	Recombinant Inbred Lines
SNP	Single Nucleotide Polymorphism
STA	starch content
SV	Structure Variation
WC	water content
XP-CLR	Cross-Population Composite Likelihood Ratio Test
