# Supplementary material for: Whole-Genome Resequencing of Cucurbita maxima and Cucurbita moschata Provides Insights into Genomic Variants Associated with Morphology and Quality Traits
Source: Int J Mol Sci. 2026 May 28;27(11):4903. doi: 10.3390/ijms27114903 (PMC13256414; doi:10.3390/ijms27114903)
Supplement: Supplementary file 1 [file ijms-27-04903-s001.zip › Supplementary Figures.pdf]

**Supplementary Figure Legends**

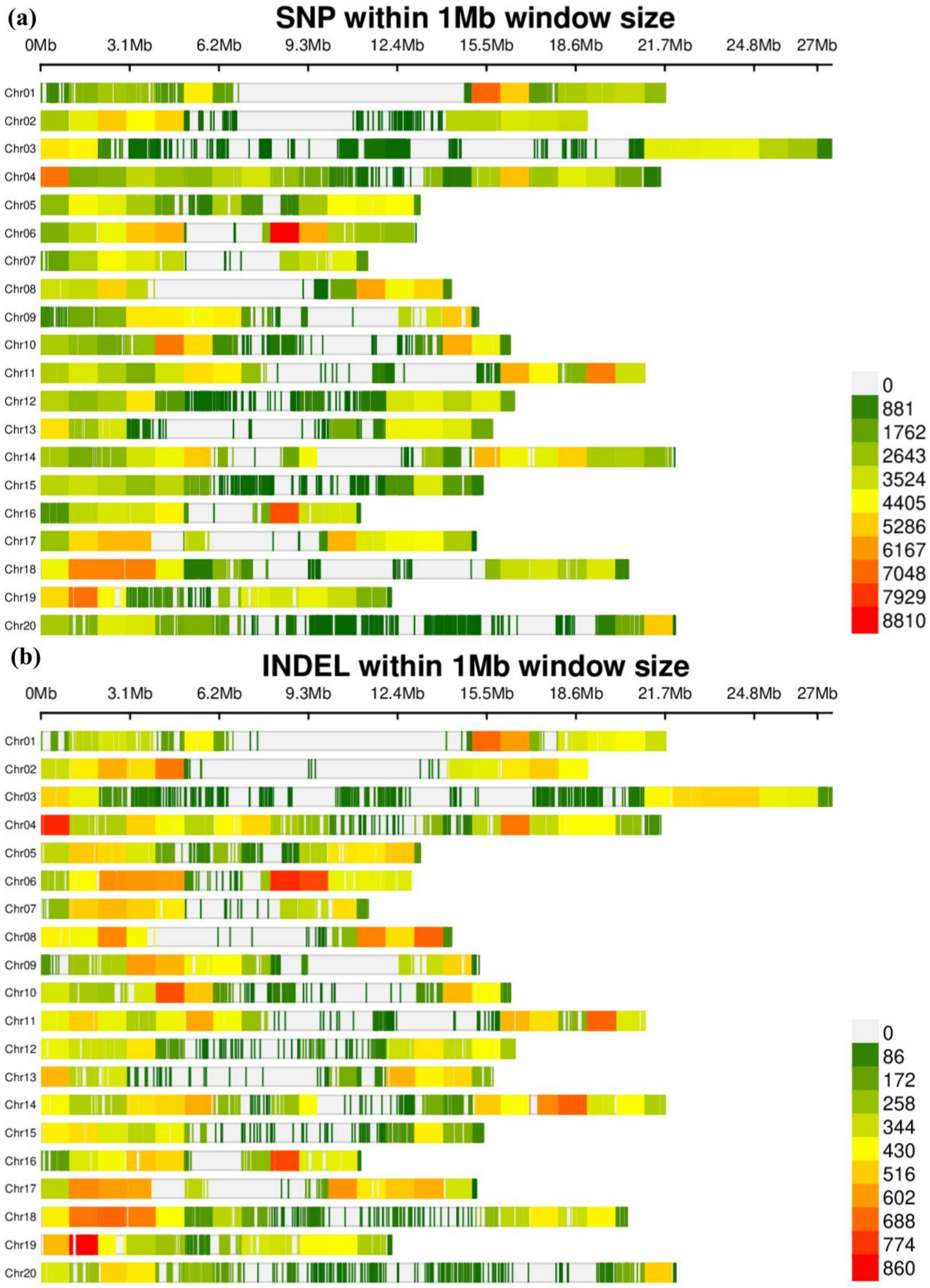


**Supplementary Figure S1:** The distrubtion of SNPs (a) and InDels (b) on the chromosomes.

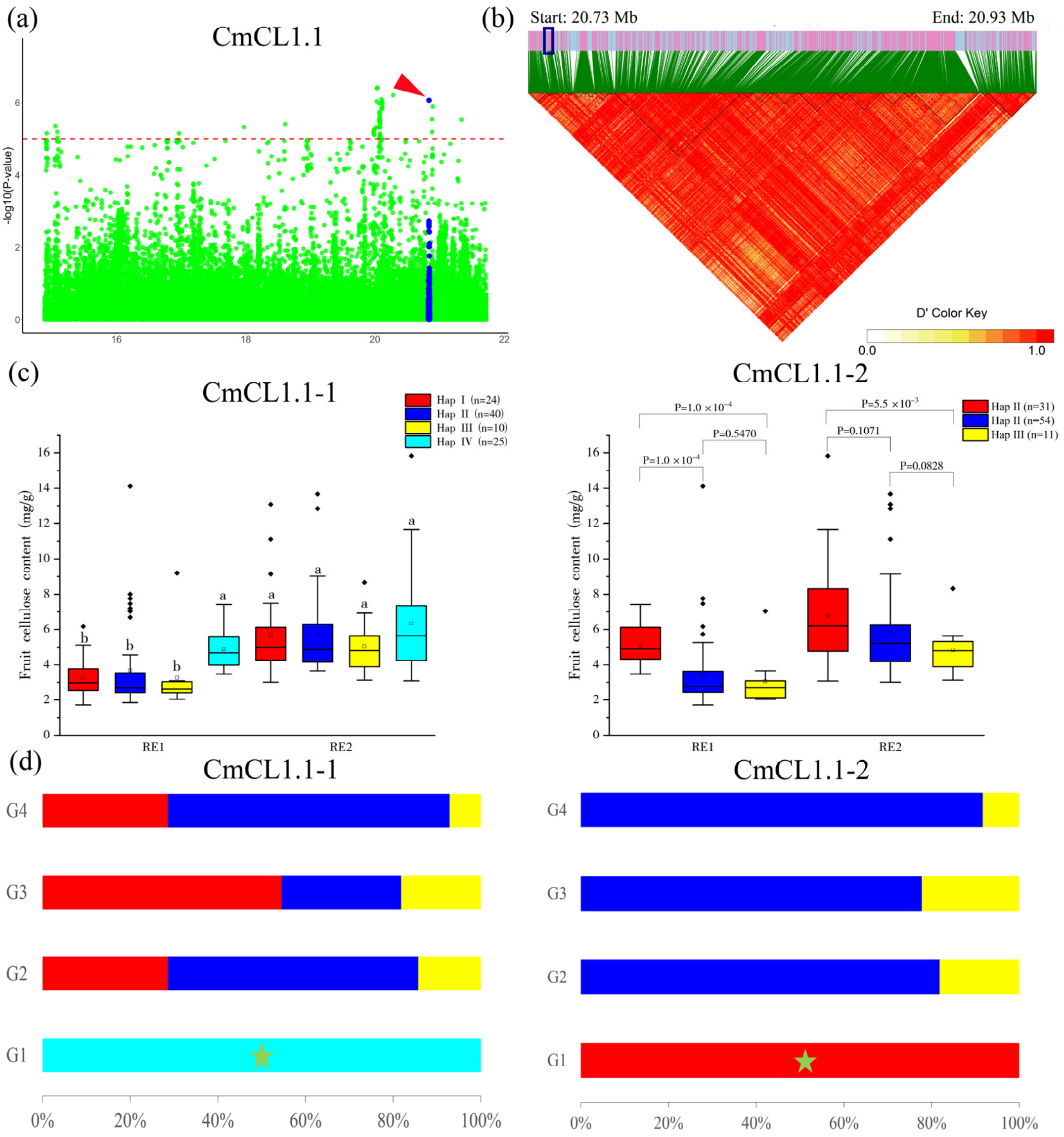


**Supplementary Figure S2:** The candidate gene selection for QTL CmCL1.1. (a): Local Manhattan plot for CmCL1.1; (b): Local linkage disequilibrium (LD) block analysis for CmCL1.1; (c): Haplotype analysis for CmCL1.1-1 and CmCL1.1-2, respectively; (d): The distribution in different pumpkin subpopulations for CmCL1.1-1 and CmCL1.1-2, respectively. Putative genes associated with signal are highlighted in blue boxes within the LD heatmaps. For haplotype analysis, the "n" values in histograms and boxplots represent the number of accessions carrying the corresponding haplotypes. In boxplots, the lower and upper edges of boxes indicate the 25% and 75% quartiles, respectively; central lines denote medians, and small hollow squares mark means. Whiskers extend to 1.5× the interquartile range, with small solid diamonds indicating outliers. P-values from two-sided Student’s t-tests are displayed above the boxplots. The oliver-green star indicates the haplotypes related to a higher fruit cellulose content.

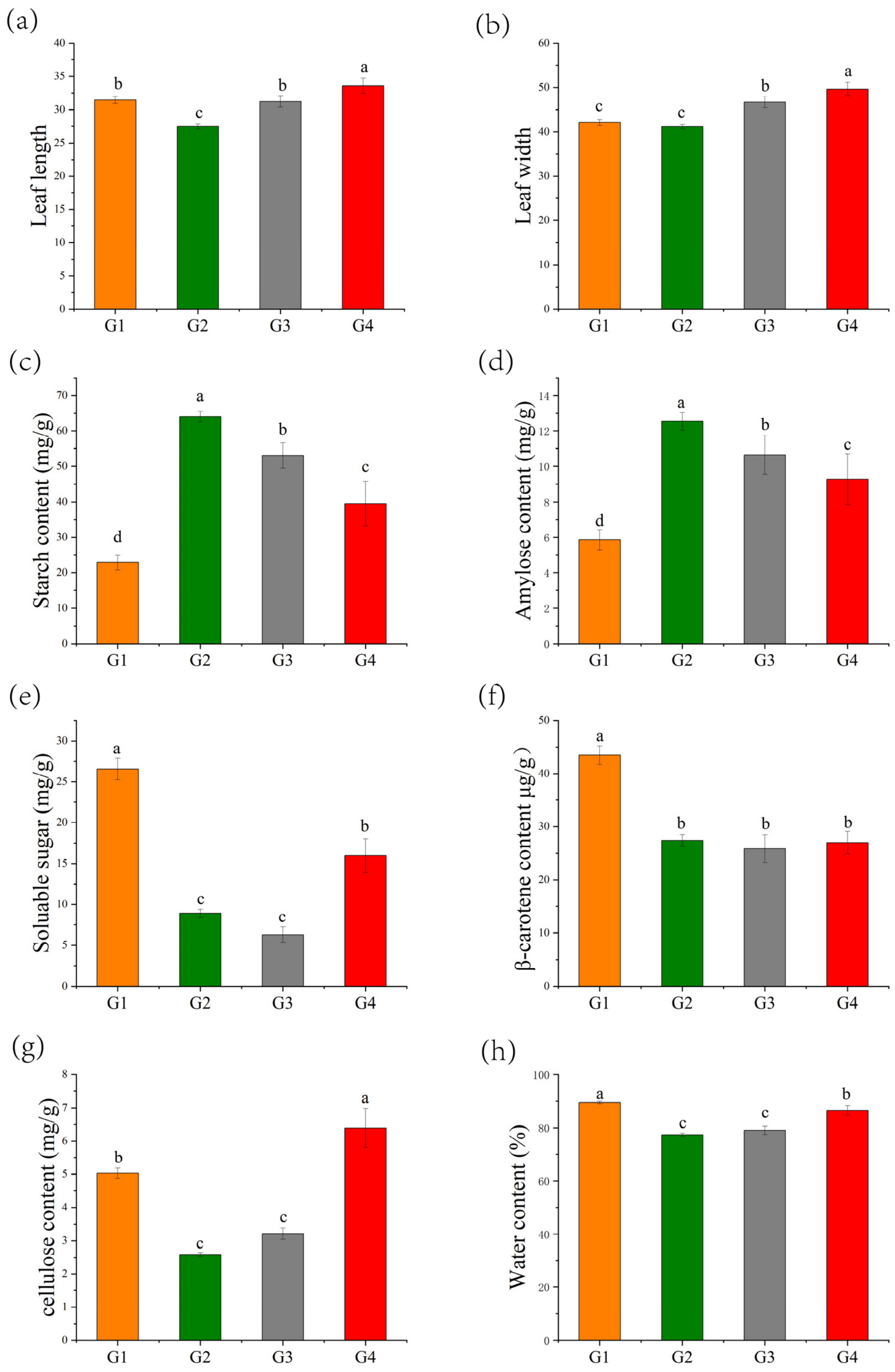


**Supplementary Figure S3:** Eight agronomic traits values among different subpopulations. (a): leaf length; (b): leaf width; (c): starch content; (d): amylose content; (e): soluble sugar content; (f): β-carotene content; (g): cellulose content; (h): water content. Mean ± SE of three replicates indicate the significant difference with Duncan test. The data followed by the same letter in the graph are not significant at $P \leq 0.05$.

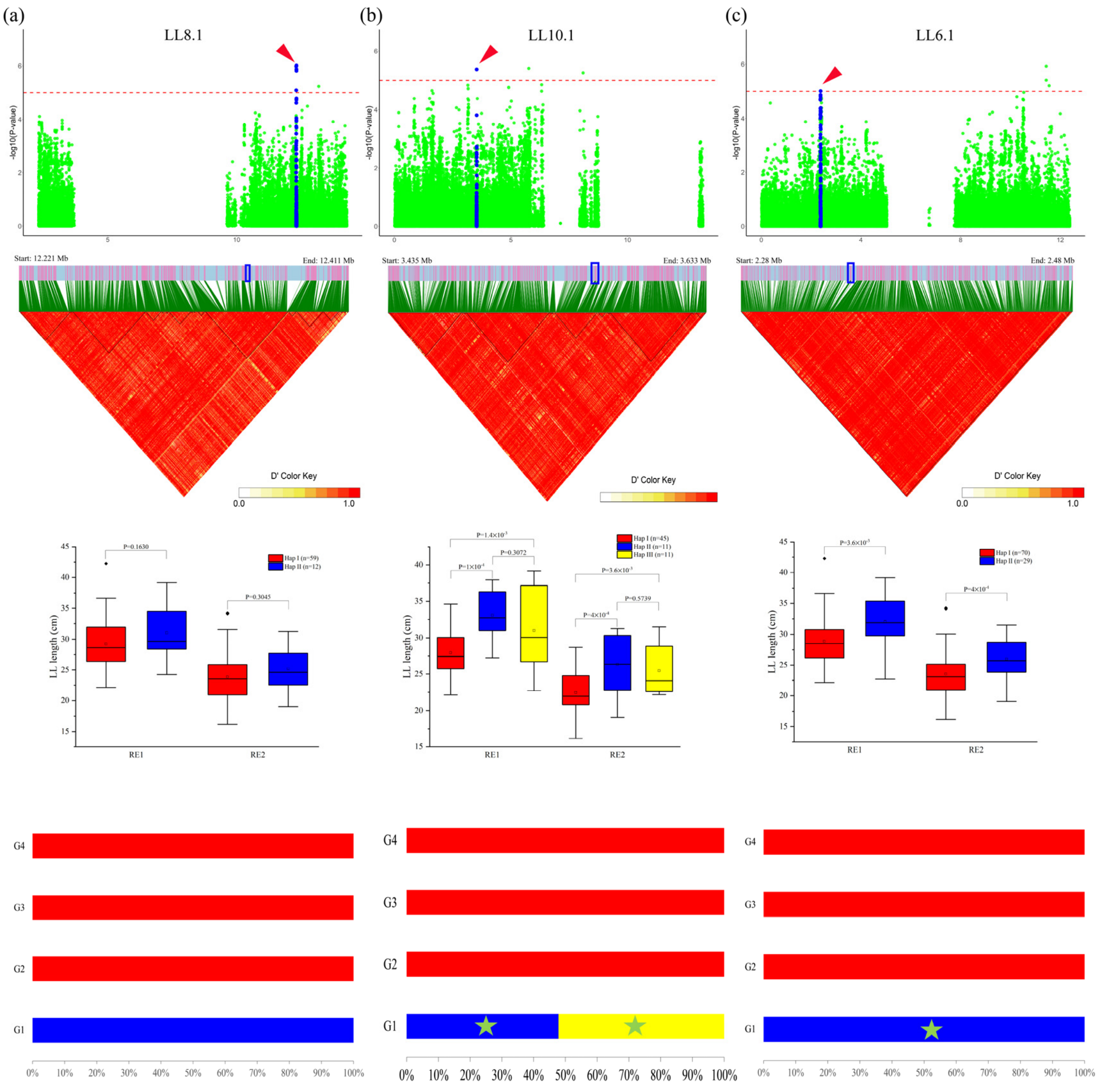


**Supplementary Figure S4:** The candidate gene selection for QTL LL8.1, LL10.1 and LL6.1. (a)-(c): Manhattan plots (top), LD block analysis (upper-middle), haplotype analysis (lower-middle) and the distribution in different pumpkin subpopulations (bottom) for LL8.1 (a), LL10.1 (b), LL6.1 (c). Putative genes associated with signal are highlighted in blue boxes within the LD heatmaps. For haplotype analysis, the "n" values in histograms and boxplots represent the number of accessions carrying the corresponding haplotypes. In boxplots, the lower and upper edges of boxes indicate the 25% and 75% quartiles, respectively; central lines denote medians, and small hollow squares mark means. Whiskers extend to 1.5× the interquartile range, with small solid diamonds indicating outliers. P-values from two-sided Student's t-tests are displayed above the boxplots. The oliver-green star indicates the haplotypes related to a longer leaf length.
